# Understanding female and male empowerment in Burkina Faso using the project-level Women’s Empowerment in Agriculture Index (pro-WEAI): a longitudinal study

**DOI:** 10.1186/s12905-021-01371-9

**Published:** 2021-06-03

**Authors:** Benjamin T. Crookston, Josh H. West, Siena F. Davis, P. Cougar Hall, Greg Seymour, Bobbi L. Gray

**Affiliations:** 1 Department of Public Health, 2137 LSB, Brigham Young University, Provo, UT 84606 USA; 2 International Food Policy Research Institute, 1201 I Street, NW, Washington, DC 20005 USA; 3 Grameen Foundation, 1400 K Street NW, Suite 550, Washington, DC 20005 USA

**Keywords:** Burkina Faso, Women’s health, Agricultural development, Women’s empowerment

## Abstract

**Background:**

Achieving gender equality and women’s empowerment is a major global priority. The purpose of this study was to determine whether the Building the Resilience of Vulnerable Communities in Burkina Faso (BRB) project, an agricultural development program, improved women’s empowerment, as measured by the project-level Women’s Empowerment in Agriculture Index (pro-WEAI).

**Methods:**

This study used a longitudinal, quasi-experimental study design. Participants included both treatment and comparison groups (total N = 751) comprising female members of savings groups and their husbands or main male household member in Burkina Faso. All participants completed the pro-WEAI questionnaire at both baseline and endline. The treatment group received a comprehensive intervention package consisting of agriculture loans and services, microenterprise loans, and education, nutrition education, and women’s empowerment programs including gender-based discussions designed to facilitate personalized changes in gender relations.

**Results:**

The proportion of the treatment group achieving empowerment did not change from baseline for women, but improved substantially for men. Women from the comparison group saw an increase in empowerment at endline while men saw a substantial decrease. Gender parity was high for women in both groups at baseline and increased slightly at endline. Women were more likely to have adequate empowerment in input in productive decisions, group membership, and membership in influential groups than men while men were more likely to have adequate empowerment in attitudes about domestic violence, control over use of income, and work balance than women. Participants from the treatment group reported an increase in the average number of empowerment indicators that they were adequate in while the comparison group saw a decrease in average adequacy over time (p = 0.002) after controlling for age, sex, and level of education.

**Conclusion:**

Despite starting at an empowerment disadvantage, the treatment group experienced gains in individual indicators of empowerment while the comparison group men and women experienced mixed results, with the women gaining, and the men losing empowerment. This research suggests that the BRB intervention may have provided some protection for the treatment group when they faced an economic down-turn prior to the endline, indicative of household resilience. Future research should consider and strengthen relationships between resilience and empowerment.

## Background

Gender equality is recognized as a universal right and efforts aimed at increasing women’s empowerment is a major global priority [1]. Kabeer defines women’s empowerment as the process by which women expand their ability to make strategic life choices, especially in situations where this ability had been denied to them previously [2]. According to Kabeer, empowerment can be achieved through the following three dimensions: (1) resources—including education, social support, and assets, (2) agency—the ability to define goals and make decisions, and (3) achievements—well-being and life outcomes that result from the use of agency.

Despite a global focus on gender equality, many persistent factors contribute to the disempowerment of women. Many nations, for example, maintain laws that disempower women by restricting travel, limiting work opportunities outside the home, dictating what types of jobs women can have, and failing to provide legal protection against sexual harassment at work. Women are consistently paid less than men for the same work. Worldwide, women earn 0.77 cents for every dollar that men earn. In sub-Saharan Africa, the gender pay gap is 31 percent for women with children, compared to 4 percent for women without children [3]. While levels of female education and literacy have improved globally, limited educational opportunities perpetuate women’s disempowerment, especially in sub-Saharan Africa where gender parity at all education levels (primary, lower secondary, and upper secondary) is far from realized [4]. According to UNESCO, 130 million girls between the ages of 6 and 17 were out of school in 2014. In addition, 15 million girls of primary school age—over half of them in sub-Saharan Africa—will never learn to read or write in primary school [5]. In 2017, women accounted for two-thirds of the world’s 750 million illiterate adults [6]. Social and cultural norms related to early marriage and childbearing similarly persist and combine to thwart empowerment efforts. Despite being considered a human rights violation, child marriage remains common with an estimated 9 percent of females marrying before the age of 15 and another 25 percent marrying between the ages of 15 and 17 [7, 8]. Child marriage is associated with early and rapid childbearing and reduced educational opportunities for the mother [9, 10]. Multiple studies have identified an association between age at marriage and intimate partner violence (IPV) or gender-based violence (GBV) [11–14]. Lack of relationship power among young brides is considered to be a key moderating factor for risk of IPV/GBV [15, 16]. The highest levels of child marriage occur collectively in sub-Saharan Africa, where 35 percent of women are married before the age of 18 [17].

Despite benefiting from historic and perpetual gender inequality and the subordination of women in the form of patriarchal privilege, some argue that men also experience disempowerment [18–20]. The very patriarchal structure and reinforcing gender norms keeping women subordinate may actually help to conceal the increasing disempowerment of men in parts of the world [21]. The socioeconomic consequences of unemployment, economic shocks, and natural disasters often effectively undermine men’s perceived social value and self-esteem [21]. As men are unable to meet social and familial expectations, they may be met with contempt from women who are left with increasing burdens of responsibility [21]. While few, if any, would argue that men are comparatively disadvantaged to women—especially in terms of the consequences to disempowerment—men in resource-poor settings experience feelings of disempowerment and are exposed to the same gender stereotypes and cultural norms contributing to women’s disempowerment. Efforts have increasingly been made to engage men in changing culturally reinforced gender stereotypes (i.e., Programme H from Promundo; Boys4Change from the Rwanda Men’s Resource Centre; HeForShe 10 × 10 × 10). Similarly, women’s empowerment programs and efforts to decrease IPV that are inclusive of both men and women in gender re-norming efforts are growing (i.e., CARE’s Cost of Violence against Women; Australia’s Male Champions of Change; Oxfam’s WE-Care). Gaining an increased understanding of the drivers of disempowerment for both women and men, and how the interplay of these contributing factors helps or hinders efforts to increase women’s empowerment is imperative.

Developing and improving comprehensive measures of empowerment is critical to measuring progress towards gender equality. Currently, several tools measure gender equality and empowerment. The Gender Gap Index (GGI), Gender Development Index (GDI), and Gender Inequality Index (GII) are global-level indices that rank countries based on the extent to which gender equality has been achieved. The GGI measures gender gaps in health, education, economy and politics [22]. The GDI assesses gender inequalities in health, knowledge, and living standards [23]. The GII measures gender gaps in reproductive health, empowerment, and economic status [24]. In addition, indices have been developed for Africa specifically. The African Gender Equality Index (AGEI) measures gender gaps in economic opportunities, human development, and law and institutions [25]. The African Gender and Development Index (AGDI) measures social power (capabilities), economic power (opportunities), and political power (agency) [26]. The Survey-based Women’s emPowERment index (SWPER) Index, which allows within-country and between-country comparisons, measures attitudes towards violence, social independence, and decision making in Africa [27]. Some studies have used Demographic and Health Survey (DHS) data from African countries to measure women’s empowerment and then performed exploratory [28] or confirmatory factor analysis [29, 30] to identify and confirm models of women’s empowerment. Additional indices measure women’s empowerment in agriculture. The Women’s Empowerment in Agriculture Index (WEAI), which relies on household surveys with men and women, measures levels and trends in women’s empowerment in agriculture at the national level [31]. The latest version of the WEAI, the project-level Women’s Empowerment in Agriculture Index (pro-WEAI), measures the impact of agricultural development interventions on women’s empowerment [32].

Agricultural development interventions present significant opportunities for expanding women’s autonomy and empowerment. Women’s empowerment influences agricultural productivity, food security, and health outcomes [33–35]. Less is known about how efforts to increase women’s empowerment may or may not impact men’s empowerment. The Building the Resilience of Vulnerable Communities in Burkina Faso (BRB) project is an integrated package of financial services, women’s empowerment, nutrition, and agricultural programs provided to community-based women’s savings group members. In particular, the BRB was designed to empower savings group members to overcome many of the social, geographic, economic, and cultural constraints that they experience during shocks and disasters. The main purpose of this study was to determine whether the BRB project improved women’s and men’s empowerment. Additionally, this study uses the pro-WEAI to examine and compare the main contributors of disempowerment for both women and men.

## Methods

### Study design and setting

This study conducted in Burkina Faso included 760 participants at baseline and 694 at endline split across treatment and comparison groups. The study is based on a longitudinal, quasi-experimental design. Sample size was determined based on an estimated attrition rate of 10% and power calculations for a 15% difference between control and treatment outcomes with 95% confidence. In May 2016, survey teams collected baseline data through interviews with participants of the treatment group and the comparison group. In November 2017, survey teams collected endline data through interviews with the same participants from both groups. The treatment group participated in the BRB project and the comparison group did not.

### Participant characteristics

The treatment group was composed of women savings group members (and their husband or main male household member) who lived in the Sanguié province of the Central-Western Region of Burkina Faso. The comparison group was composed of women savings group members (and their husband or main male household member) who lived in the Nayala province of the Boucle de Mouhoun Region of Burkina Faso. While the comparison group was selected because of proximity and general community similarities, there were key differences between the two groups at the individual level.

### Description of treatment

An integrated set of financial services, women’s empowerment, nutrition, and agricultural programs were provided to savings group members. Women farmers received support from local agricultural extension managers to assist in caring for crops and livestock. Trained staff facilitated learning conversations on gender dialogues, agriculture, and nutrition to encourage group members, their partners, and their communities to develop their own visions for change in gender relations, particularly as they related to land use for home gardens. Local financial organizations participating in the BRB project provided loan products (agriculture loans, microenterprise loans, and group loans) and ongoing support to SG members. Additional program details can be found elsewhere [36].

### Survey instrument

The International Food Policy Research Institute developed the pro-WEAI to measure the impact of agricultural development interventions on women’s empowerment. The quantitative and qualitative pro-WEAI instruments are open access and available elsewhere [37]. The pro-WEAI was translated into French and piloted in Burkina Faso in April 2016. The adapted version of pro-WEAI used in this study is based on 11 indicators. For each indicator, respondents are classified as adequate (= 1) or inadequate (= 0) based on predetermined thresholds. Pro-WEAI is composed of two sub-indices: the Three Domains of Empowerment sub-index (3DE), which measures the extent and depth of women’s empowerment, and the Gender Parity sub-index (GPI), which measures gender parity between women and men in the same household based on their respective empowerment scores.

To assess women’s and men’s empowerment, we calculate (i) the individual’s empowerment score, defined as the sum of the 11 pro-WEAI indicators; and (ii) the individual’s empowerment status, which classifies an individual as empowered (= 1) if he or she achieves adequacy in at least 9 of the 11 indicators. To assess gender parity, we calculate (i) the intrahousehold inequality score, defined as the difference in the empowerment scores between the woman and her partner and equal to 0 if the woman is empowered; and (ii) the household’s gender parity status, which classifies a household as achieving gender parity (= 1) if the woman is empowered or if her empowerment score is at least as high as the empowerment score of her partner. Table 1 outlines the 11 indicators used in the pro-WEAI for this study and describes the determination of adequacy for each indicator.

**Table 1:** Indicators and measures of adequacy

**Autonomy in income**
A Relative Autonomy Index (RAI) score was calculated by summing responses to three vignettes about a person’s motivation for how they use income generated from agricultural and non-agricultural activities. A participant was considered adequate in autonomy of income if they were more motivated by their own values than by coercion or fear of others’ disapproval
**Attitudes about intimate partner violence against women**
Believes husband is NOT justified in hitting or beating his wife in all 5 scenarios: 1) She goes out without telling him 2) She neglects the children 3) She argues with him 4) She refuses to have sex with him 5) She burns the food
**Respect among household members**
Meets ALL of the following conditions related to their spouse, the other respondent, or another household member: 1) Respondent respects relation (MOST of the time) AND 2) Relation respects respondent (MOST of the time) AND 3) Respondent trusts relation (MOST of the time) AND 4) Respondent is comfortable disagreeing with relation (MOST of the time)
**Input in productive decisions**
Meets at least ONE of the following conditions for ALL of the agricultural activities they participate in 1) Makes related decision solely, 2) Makes the decision jointly and has at least some input into the decisions 3) Feels could make decision if wanted to (to at least a MEDIUM extent)
**Ownership of land and other assets**
Owns, either solely or jointly, at least ONE of the following: 1) At least THREE small assets (poultry, non-mechanized equipment, or small consumer durables) 2) At least TWO large assets 3) Land
**Access to and decisions on financial services**
Meets at least ONE of the following conditions: 1) Belongs to a household that used a source of credit in the past year AND participated in at least ONE sole or joint decision about it 2) Belongs to a household that did not use credit in the past year but could have if wanted to from at least ONE source 3) Has access, solely or jointly, to a financial account
**Control over use of income**
Has input in decisions related to how to use BOTH income and output from ALL of the agricultural activities they participate in AND has input in decisions related to income from ALL non-agricultural activities they participate in, unless no decision was made
**Work balance**
Works less than 10.5 h per day: Workload = time spent in primary activity + (1/2) time spent in childcare as a secondary activity
**Visiting important locations**
Meets at least ONE of the following conditions: 1) Visits at least TWO locations at least ONCE PER WEEK of [city, market, family/relative], or 2) Visits least ONE location at least ONCE PER MONTH of [health facility, public meeting]
**Group memberships**
Active member of at least ONE group
**Membership in influential groups**
Active member of at least ONE group that can influence the community to at least a MEDIUM extent

### Statistical analysis

Frequency statistics were calculated and presented separately for treatment and comparison groups at both baseline and endline. Because the treatment and comparison groups were not similar at baseline, Differences-in-differences (DiD) modeling was used to estimate the impact of the BRB intervention on women’s empowerment (PWI) after controlling for gender, age, and level of education. DiD estimates the differential effect of the treatment by calculating the average change in women’s empowerment in the treatment and comparison groups from baseline to endline. This approach attempts to approximate an experimental design method by adjusting for differences in the outcome at baseline between comparison and treatment groups.

## Results

Male respondents from the treatment group were significantly older than the comparison group male respondents (Table 2). Ever attended school and the proportion of respondents that were female were similar for both groups. While there was some loss to follow-up in both groups, demographic differences remained similar to baseline. Similarly, baseline demographics among the full BRB program evaluation survey show significant differences between the treatment and comparison groups [38]. The treatment group was ethnically more Gourounsi and Christian and less well-off economically and more food insecure, while the comparison group was more Mossi, Muslim, better-off economically, and more food secure.

**Table 2:** Key demographics from respondents by treatment

Indicator	Treatment Female (n = 191)	Comparison Female (n = 189)	*P* value	Treatment Male (n = 191)	Comparison Male (n = 189)	*P* value
Mean age in years (SD, min, max)	43.5 (11.8, 21, 76)	42.1 (11.2, 18, 68)	.2633	57.6 (14.3, 26, 93)	51.3* (13.2, 23, 91)	.001
Ever attended school (%)	17.4	13.9	.375	18.5	20.8	.597

At baseline, 38 percent of women and 36 percent of men in the treatment group were identified as empowered, compared to 44 percent of women and 75 percent of men in the comparison group (Table 3). At endline, the proportion of the treatment group achieving empowerment did not change from baseline for women, but improved substantially for men (47%). Women from the comparison group saw an increase in empowerment at endline (51%) while men saw a substantial decrease (67%). Gender parity was high for households in both groups at baseline and increased slightly at endline. The intrahousehold inequality score was 26 percent for households in the treatment group and 21 percent for households in the comparison group. Both groups saw a slight decrease in the gap at endline.

**Table 3:** pro-WEAI results by gender, treatment, and time

	Baseline				Endline			
	Treatment (%)		Comparison (%)		Treatment (%)		Comparison (%)	
Indicator	Female	Male	Female	Male	Female	Male	Female	Male
Empowerment Score	0.69	0.71	0.76	0.89	0.72	0.77	0.79	0.86
Achieving empowerment (%)	38	36	44	75	38	47	51	67
Gender Parity Index	0.88		0.90		0.90		0.93	
Average empowerment gap	0.26		0.21		0.25		0.20	

Respondents with missing indicators are dropped from the sample

Adequacy for each pro-WEAI indicator varied by gender, group, and time (Table 4). For example, women were more likely to be adequate in input in productive decisions, group membership, and membership in influential groups. Men were more likely to be adequate in attitudes about domestic violence, control over use of income, and work balance. Adequacy in attitudes about domestic violence improved for all groups over time while control of use of income decreased over time for all groups. Men and women across both the treatment and comparison groups lost adequacy in control over use of income at endline compared to baseline. While men and women in the treatment group lost adequacy in autonomy in income, men and women in the comparison group gained adequacy in autonomy in income. Women and men in the treatment group lost adequacy in work balance, women notably so, while men and women in the comparison group gained adequacy.

**Table 4:** Adequacy status for PRO-WEAI indicators by sex, treatment, and time

	Baseline				Endline			
	Treatment (%)		Comparison (%)		Treatment (%)		Comparison (%)	
Indicators	Female	Male	Female	Male	Female	Male	Female	Male
Autonomy in income	62.8	60.7	44.4	66.1	30.7	48.2	58.2	69.6
Attitudes about domestic violence	37.7	67.0	22.2	57.7	48.9	70.8	52.2	75.6
Respect among household members	89.5	91.6	95.2	94.7	97.1	99.4	96.0	99.4
Input in productive decisions	33.0	5.2	46.0	32.8	47.7	13.1	39.6	22.6
Ownership of land and other assets	88.5	99.0	96.8	100	97.2	100	98.4	100
Access / decisions on credit / finance	8.9	11.5	13.2	21.7	18.2	16.1	8.2	19.1
Control over use of income	62.3	91.6	67.7	95.8	39.2	71.4	37.4	62.5
Work balance	62.8	77.5	54.5	87.3	37.5	69.6	75.8	91.1
Visiting important locations	78.0	79.1	92.1	87.3	86.4	86.3	91.8	80.4
Group membership	75.4	58.1	97.9	91.5	98.9	78.0	95.1	82.1
Membership in influential groups	70.2	54.5	95.8	90.5	86.9	70.8	94.5	80.4

Among those classified as disempowered, the drivers of disempowerment remained similar for both the treatment and comparison groups (for both men and women) over time (Table 5). The main drivers included access and decisions on credit and finance, input in productive decisions, autonomy in income, and attitudes about domestic violence. Membership in influential groups was a larger driver at baseline than endline for treatment groups and was more likely to contribute to male disempowerment for both groups. Control over use of income as a contributor to disempowerment increased in all groups over time and was more likely to contribute to female disempowerment. Attitudes about domestic violence were larger contributors to disempowerment for women than men and decreased slightly over time.

**Table 5:** Contributors to disempowerment by sex, treatment, and time

	Baseline				Endline			
	Treatment (%)		Comparison (%)		Treatment (%)		Comparison (%)	
Indicators	Female	Male	Female	Male	Female	Male	Female	Male
Autonomy in income	8.3	9.5	13.5	14.2	15.5	15.1	11.4	8.3
Attitudes about domestic violence	14.3	7.8	18.5	14.2	12.7	8.5	13.9	9.8
Respect among household members	2.9	2.7	1.4	3.2	0.9	0.2	1.5	0.4
Input in productive decisions	14.9	19.9	16.3	17.8	14.4	19.5	18.5	18.9
Ownership of land and other assets	3.2	0.3	1.0	0.0	0.9	0.0	0.7	0.0
Access / decisions on credit / finance	17.4	18.9	19.3	20.5	17.0	19.1	20.4	19.3
Control over use of income	10.0	2.2	11.6	2.3	15.1	7.8	17.8	9.4
Work balance	8.5	5.6	13.5	4.1	15.1	6.8	8.5	3.1
Visiting important locations	5.1	6.0	2.6	9.1	3.7	4.5	3.2	9.1
Group membership	6.9	13.0	0.8	6.8	0.4	8.2	1.9	10.2
Membership in influential groups	8.5	14.1	1.4	7.8	4.1	10.4	2.2	11.4

Differences-in-differences (DID) modeling was used to estimate the change in adequacy across the 11 empowerment indicators as a result of the BRB intervention (Table 6). Participants from the treatment group reported an increase in the average number of empowerment indicators that they were adequate in while the comparison group saw a decrease in average adequacy over time (p = 0.002) after controlling for age, sex, and level of education.

**Table 6:** Difference-in-differences in adequacy

Time	Treatment mean (SD)	Condition	
		Comparison mean (SD)	Difference
Baseline	6.82 (1.68)	7.76 (1.46)	0.94
Endline	7.03 (1.46)	7.61 (1.45)	0.58
Change	0.21	− 0.15	0.36*

^*^p = 0.002, controls for sex, age and education

## Discussion

Notwithstanding an economic downturn resulting from a significant drought and subsequent poor harvest that occurred at the time of the endline survey [36], this study provides valuable insights related to women’s and men’s empowerment. Results of the pro-WEAI reveal that women in the comparison group experienced greater improvements in empowerment over time. Results for men were remarkably different: men in the treatment group experienced an improvement in empowerment while men in the comparison group experienced a substantial decline in empowerment.

Women in both the treatment and comparison groups gained in gender parity over time; however, the gain experienced by the comparison group may have been affected by men’s loss of empowerment. Men and women in the treatment group started out with an empowerment disadvantage, compared to the comparison group, and maintained this disadvantage at the endline. This is consistent with findings from the full BRB program evaluation baseline which found that the treatment group also started out with a disadvantage in economic outcomes and food security [36]. However, it is important to note that when assessing individual adequacy scores and adjusting for baseline, sex, age, and education, results indicate the treatment group made greater gains in individual indicators of empowerment, even though this did not result in them passing the thresholds for classifying them as empowered.

Regions where the study took place experienced a significant economic downturn due to a drought resulting in poor harvests. While much of the literature on gender and climate change suggest that women are disproportionately and negatively impacted by climate-change or weather events compared to men [39], these results suggest that some interventions geared toward women may be protective of men as well. Men in the comparison group may have lost some confidence in their ability to withstand this downturn. The engagement of women in both the treatment and comparison groups in the savings groups may have contributed to sustained or improved empowerment.

Though the relationship between male empowerment and household economics is not well documented, global patterns of male participation in the labor force demonstrate income generating obligations predominantly are carried out by men [40]. Further, many men see their primary societal role as providing for their respective household [41]. Hence, men’s self-esteem is often tied directly to employment as noted by ProMundo’s State of the World’s Fathers 2019 report. Not surprisingly then, when men are underemployed, risk of GBV for women in the household increases [42, 43].

Some key drivers of disempowerment were similar across time, groups, and sex. For example, decisions on credit and finance, input in productive decisions, and autonomy in income were major contributors of disempowerment for all groups and times. These findings suggest that household economics contributed most to the disempowerment of this population. This is consistent with other qualitative and quantitative results, both from the qualitative assessment conducted for the BRB project [44] and elsewhere [32, 45–47].

Women participating in savings groups alone are more likely to see increased incomes and savings, economic independence [48], better balancing of spending and saving, improved or varied livelihood activities [49], and better commercial results [50]. In short, participation in savings groups positively impacts women’s economic, social, and political empowerment. Finally, it is important to note that savings groups have also not been found to adversely affect reports of domestic violence [51].

Contrary to research that indicates men have more decision-making power and access to resources [52, 53], men were also found to be disempowered in decisions on credit and finance, input in productive decisions, and autonomy in income. In 2017, the World Bank reported a 17 percentage-point gender gap between women’s and men’s access to bank accounts in Burkina Faso; 51 percent of men had a bank account compared to 34 percent of women [52]. Men and women borrowed from financial institutions at a similarly low rate of 9 percent. In this study, women’s disempowerment in the treatment group was driven slightly less by access to financial services when compared to their spouses at the endline [36].

Women’s access to credit, in particular, is fraught with challenges. Though it increases opportunities for economic improvement for women, it also can lead to anxiety and struggle, especially in situations where women may carry repayment responsibility though they may not actually make decisions on the use of the loan. This can be addressed, at least in part, through savings groups that give women opportunities to protect their money from partners [54].

While some contributors to disempowerment were similar for men and women, some contributors did differ by sex. For example, attitudes about GBV were more likely to contribute to female disempowerment. That is to say that women in this study were more likely than men to indicate a husband is justified in hitting or beating his wife for going out without telling him, neglecting the children, arguing with him, refusing to have sex with him, or burning the food. These results are similar to findings from previous research on the pro-WEAI [32] and the literature [55] from numerous Sub-Saharan African countries where women were more likely to rationalize GBV than men. While increasing women’s own condemnation of GBV is an important attitudinal shift, research on strategies to reduce GBV have found that efforts to influence the intra-household distribution of economic resources, promotion of gender equitable norms, promotion of joint decision-making and increasing coverage of messages (such as through media) to the general public regarding alternatives to violence as a means to resolve conflict are strategies proven to reduce the likelihood of GBV [56–60]. Peacock and Barker note that one way to address GBV is by targeting men through women’s economic empowerment initiatives [61]. A study conducted in Rwanda by CARE-Rwanda and ProMundo compared a control group (savings groups of women where men were not deliberately included) to savings groups of men-only and savings groups where couples were engaged and found that engaging men led to more equitable household decision-making, decreased couple conflict, increased communication, and higher income gains for families [62]. Peacock and Barker also suggest that policies aimed at ending GBV by engaging men should 1) promote human rights, including rights of women and girls; 2) remain accountable to and in dialogue with women’s rights movements and organizations, 3) enhance men’s and boy’s lives, 4) be inclusive and responsive to diversities among men, and 5), address the social and structural determinants of gender inequalities [61].

### Implications, recommendations, and future research

The pro-WEAI index works to measure empowerment by efficiently considering a number of multifaceted factors while also allowing researchers, evaluators, and practitioners to understand the contributions of individual factors towards establishing empowerment. The results from this assessment leveraging the pro-WEAI suggest that future programs that aim to improve women’s economic empowerment should ensure a meaningful engagement of men, particularly as it relates to the formation of savings groups’ access and use of credit, as well as to address GBV. Men need to feel they have a seat at the table as well as a positive role in the empowerment of their wives, daughters, and other women close to them.

Given the concern of financial abuse or increase of stress carried by women when they carry the household debt, future iterations of the pro-WEAI may seek to balance the current focus on access to and use of credit with the responsibility for repayment and the stress they feel carrying this responsibility. While savings groups and microfinance-based strategies often focus on new product or channel development with little recognition of the role that stresses and shocks related to GBV or conflict and instability at the household or community levels play in the lives of poor women, this is an important area for further innovation.

Addressing GBV requires not only addressing those who perpetrate it (men), but also those who justify it (more often women). Incorporating approaches that have been shown to mitigate the risk of and decrease gender-based violence should be scaled up, including approaches such as gender and community dialogues and approaches that increase intra-household distribution of economic resources and cooperation. Finally, women’s empowerment cannot come at the disempowerment of men. While gender equality assumes women need to “catch up” it is also possible that men can “fall back” to the detriment of women and men alike. Particularly among poor populations, as has been shown here, men are almost equally disempowered. Additional research is needed to understand the negative consequence of male empowerment, not just on themselves, as is often done for women, but on the household as a whole, particularly since a key risk of potential male disempowerment is the resort to GBV as a stronghold on control of the resources and people that surround a male primary income earner.

Our study had some limitations. First, the noted economic downturn at the endline posed a challenge to this study and results have to be interpreted with this limitation in mind. Another round of data collection for the BRB program evaluation, that was unable to include the pro-WEAI, did occur one year after the pro-WEAI endline and the results were suggestive of much stronger impacts for the treatment group, particularly as they bounced back from the economic downturn [37]. Second, while it was originally planned that the full BRB program evaluation would be integrated with the pro-WEAI survey to provide additional demographic and household data, this was not possible due to lack of consistent household identifiers. Hence, the main controls (age, gender, and education) used in the analysis were the only relevant controls available. A more complete appraisal of the differences between the comparison and treatment groups can be found in the full evaluation report [36]. Next, there were key differences between the comparison and treatment groups. While analytical methods were chosen to account for these differences, making comparisons must be considered in the context of these key differences. Finally, all women participating in this study were members of self-help groups that also may have received other non-financial services before or during the program period. Women’s very membership in savings groups also receiving other interventions may also cloud the ability to tease out the impacts of the BRB program’s agricultural, nutrition, and women’s empowerment interventions.

## Conclusion

The pro-WEAI data suggests that men and women of the treatment group experienced statistically significant gains in adequacy across individual pro-WEAI indicators, even though this did not translate into passing thresholds to be considered improvements in empowerment. While women in the comparison group saw gains in empowerment, men in the comparison group experienced losses in empowerment. This research suggests that the BRB intervention may have provided some protection for men and women in the treatment group when they faced an economic down-turn prior to the endline, indicative of household resilience. Leder suggests that there may be a relationship between empowerment and resilience, particularly once the extent to which each dimension or indicator of empowerment influences resilience is determined. This is an opportunity for future research [63].

To understand the influence of a multiple-intervention project designed to influence women’s economic empowerment, the pro-WEAI has been found to be a useful tool for identifying priorities for improving empowerment suggesting a “stay the course” for some already-implemented interventions such as the expansion of agriculture and income-generating activity credit and an expansion of other interventions such as the gender dialogues to take a stronger emphasis on attitudes towards GBV.

## Data Availability

The datasets used during the current study are available from the corresponding author on reasonable request.

## Abbreviations

3DE: Three Domains of Empowerment sub-index
AGDI: African Gender and Development Index
AGEI: African Gender Equality Index
BRB: Building the Resilience of Vulnerable Communities in Burkina Faso
DiD: Difference-in-Difference
DHS: Demographic and Health Survey
GBV: Gender-based violence
GDI: Gender Development Index
GGI: Gender Gap Index
GII: Gender Inequality Index
GPI: Gender Parity sub-index (GPI)
IPV: Intimate partner violence
pro-WEAI: Project-level Women’s Empowerment in Agriculture Index
RM-TWG: Resilience Measurement Technical Working Group
SWPER: Survey-based Women’s emPowERment index
WEAI: Women’s Empowerment in Agriculture Index
